# *Wolbachia* in European Populations of the Invasive Pest *Drosophila suzukii*: Regional Variation in Infection Frequencies

**DOI:** 10.1371/journal.pone.0147766

**Published:** 2016-01-25

**Authors:** Julien Cattel, Rupinder Kaur, Patricia Gibert, Julien Martinez, Antoine Fraimout, Francis Jiggins, Thibault Andrieux, Stefanos Siozios, Gianfranco Anfora, Wolfgang Miller, Omar Rota-Stabelli, Laurence Mouton

**Affiliations:** 1 Université de Lyon, Université Lyon1, Laboratoire de Biométrie et Biologie Evolutive, UMR CNRS 5558, 43 Bd du 11 Novembre 1918, 69622 Villeurbanne Cedex, France; 2 Chemical Ecology lab, Department of Sustainable Agro-ecosystems and Bio-resources, Fondazione Edmund Mach, San Michele all'Adige (TN), Italy; 3 Laboratory of Genome Dynamics, Department of Cell and Developmental Biology, Center for Anatomy and Cell Biology, Medical University of Vienna, Vienna, Austria; 4 Department of Genetics, University of Cambridge, Cambridge, United Kingdom; 5 Institut de Systématique, Évolution, Biodiversité ISYEB - UMR 7205 – CNRS, MNHN, UPMC, EPHE Muséum national d’Histoire naturelle, Sorbonne Universités57 rue Cuvier, CP50F-75005, Paris, France; 6 Institute of Integrative Biology, University of Liverpool, Liverpool, United Kingdom; University of Innsbruck, AUSTRIA

## Abstract

The invasive pest *Drosophila suzukii* is characterized by a specific fresh-fruit targeting behavior and has quickly become a menace for the fruit economy of newly infested North American and European regions. *D*. *suzukii* carries a strain of the endosymbiotic bacterium *Wolbachia*, named *w*Suz, which has a low infection frequency and no reproductive manipulation capabilities in American populations of *D*. *suzukii*. To further understand the nature of *w*Suz biology and assess its utility as a tool for controlling this pest’s populations, we investigated the prevalence of *Wolbachia* in 23 European *D*. *suzukii* populations, and compared our results with those available in American populations. Our data showed a highly variable infection frequency with a mean prevalence of 46%, which is significantly higher than the 17% found in American populations. Based on Multilocus Sequence Typing analysis, a single *w*Suz strain was diagnosed in all European populations of *D*. *suzukii*. In agreement with American data, we found no evidence of cytoplasmic incompatibility induced by *w*Suz. These findings raise two questions: a) why *Wolbachia* is maintained in field populations of *D*. *suzukii* and b) what are the selective forces responsible for the variation in prevalence within populations, particularly between European and American continents? Our results provide new insights into the *D*. *suzukii-Wolbachia* association and highlight regional variations that await further investigation and that should be taken into account for using *Wolbachia*-based pest management programs.

## Introduction

*Drosophila suzukii* Matsumura (Diptera: Drosophilidae), first described in Japan in the early 1900s [1], is an invasive pest of Southeast Asian origin. Since its early detection in California (USA), Spain and Italy (Europe) in 2008, *D*. *suzukii* has rapidly spread through these two continents aided by global trading and absence of niche competitors [2,3,4,5,6]. Although the vast majority of *Drosophila* species are not fruit pests (larvae developing only in damaged or rotten fruits), *D*. *suzukii* is able to lay eggs on healthy ripening fruits thanks to female's serrated ovipositor [7]. A wide range of soft and stone fruits including raspberry, strawberry, blueberry, plums and grapes come under *D*. *suzukii’*s damage range [4,8]. Damage is caused by developing larvae inside the fruit, leading to millions of dollars of annual economic losses worldwide [9,10]. Control of *D*. *suzukii* populations in the field mainly relies on the use of chemical pesticides, a practice with serious drawbacks because of its use close to harvest and the consequent risk of high residues left on fruits. Management agendas are therefore in permanent search for alternative strategies including those based on bio-control [4,11,12,13,14,15].

Previous studies revealed the presence of the bacterium *Wolbachia* in *D*. *suzukii* (strain named *w*Suz) [16,17,18,19,20] but of no other heritable bacterial symbionts [21]. This is not surprising since *Wolbachia* is widespread among terrestrial arthropods with an estimate of 52% of species infected at variable prevalence [22]. *Wolbachia* is a maternally inherited bacterium that has established a wide range of relationships with their hosts, from mutualism to parasitism. It is particularly known for its ability to manipulate host reproduction through different strategies to maximize its spread and maintenance in host populations [23]. The most common strategy is cytoplasmic incompatibility (CI), a sperm-egg incompatibility expressed in crosses between infected males and uninfected females leading to the death of embryos in diploid species, thereby facilitating *Wolbachia* to spread throughout host populations [24,25]. However, in the absence of CI or other means of reproductive manipulation, *Wolbachia* can also provide direct host fitness benefits (e.g. increasing survival time, enhancing reproduction, provisioning nutrition or protection against viruses), which may sometimes allow infected individuals to outcompete uninfected counterparts [26]. For example, in *D*. *mauritiana*, *Wolbachia* infection leads to a decrease of apoptosis and an increase of mitotic divisions in germ line stem cells of infected female ovaries, resulting in more eggs produced than in uninfected ovaries [27]. Lack of any of the suggested effects on the host can potentially lead to loss of infection from a population [28,29,30]. Overall, the infection dynamics of *Wolbachia* depend on the bacterial strain, host species, genetic background, environmental conditions and interactions among these factors [31]. Recently, a field survey in North America indicated a low prevalence of *Wolbachia* in field populations of *D*. *suzukii* from four localities sampled over two years (prevalence ranged between 7 to 58%), with a mean infection rate of 17% [19]. This study showed no signs of CI or any other reproductive phenotype induced by *w*Suz in *D*. *suzukii*. Moreover, the imperfect transmission of *Wolbachia* from wild-caught females to their offspring (20 to 95%) suggested possible direct fitness benefits provided by *w*Suz to its American *D*. *suzukii* host but the phenotypic nature of these benefits has not yet been determined.

In order to improve our understanding of the global infection dynamics of *w*Suz in natural *D*. *suzukii* populations and thereby assess the utility of *Wolbachia* as a tool for controlling this insect pest population in the field, we investigated the prevalence of *Wolbachia* in 23 localities among eight European countries, typed *Wolbachia* strains through a Multilocus Sequence Typing approach and performed mating experiments on two different European populations.

## Materials and Methods

### Field sampling

**Fig 1:**
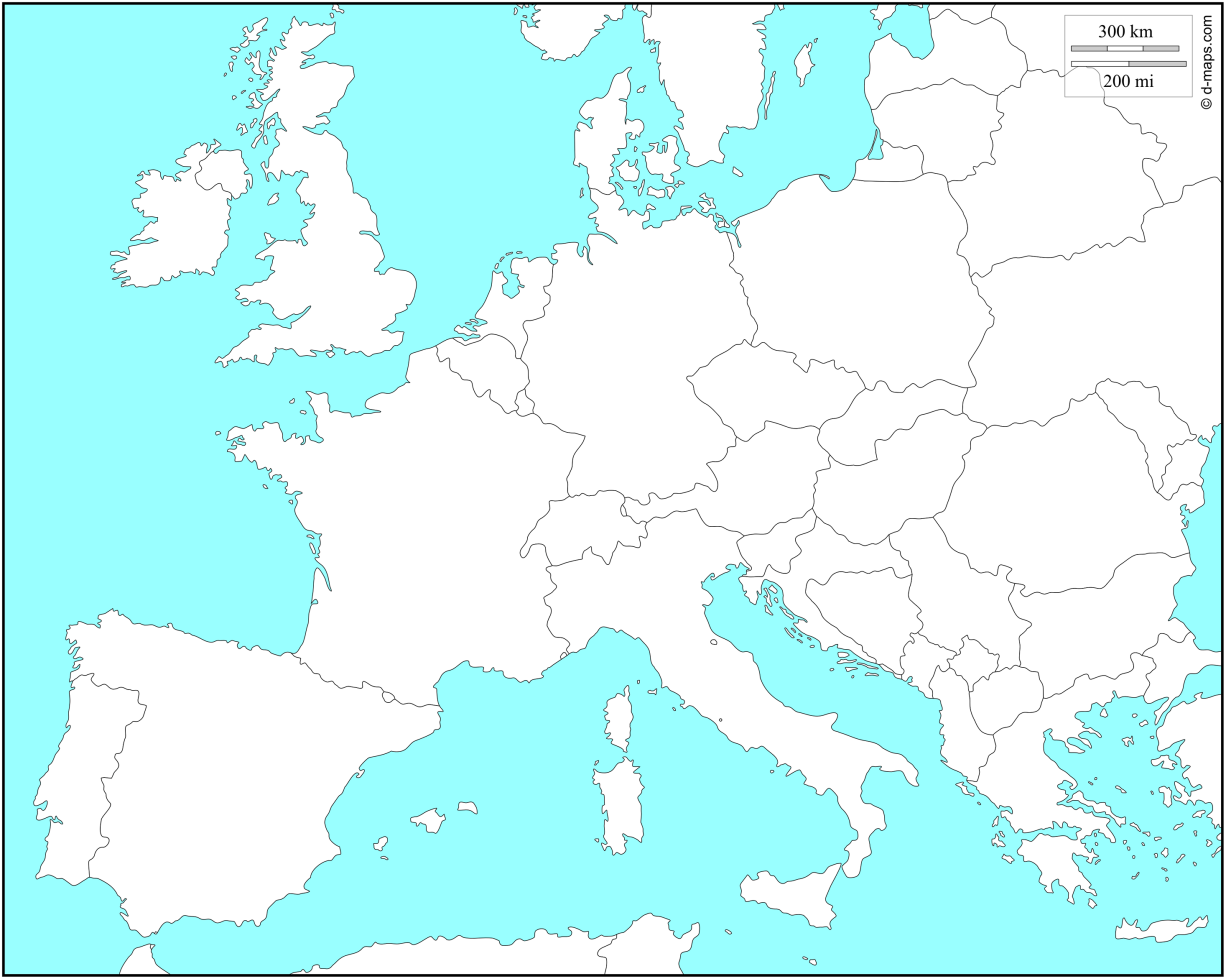
*Wolbachia* prevalence and collection sites for *D*. *suzukii* individuals. A number was assigned for each sampling site and details for each locality are given in the Table 1. Reprinted from http://d-maps.com under a CC BY license, with permission from Daniel Dalet, original copyright 2007–2016.

561 individuals were sampled between 2010 and 2014 in 23 locations from eight different countries (Austria, France, Germany, Italy, Slovenia, Spain, Switzerland and United Kingdom) of the European continent (Fig 1 and Table 1). Adults were caught directly in orchards or from attractive traps, placed alive in ethanol (96%) and stored at -20°C until DNA extraction. For *Wolbachia* screening, we gave priority to females because they are known to be responsible for *Wolbachia* transmission.

**Table 1:** Sampling information and percentage of individuals infected by *Wolbachia*.

	Country	Locality	Collection period	Total No. (M+F)	No. of infected individuals	Infection rate (95% CI)
1	Austria	Neustift am Walde	Oct, 2014	19 (6+13)	12	0.63 (0.38–0.84)
2	United Kingdom	East Malling	Sep, 2014	24 (6+18)	4	0.17 (0.05–0.37)
3	France	Montauban	Aug, 2010	9 (7+2)	4	0.44 (0.14–0.79)
4	France	Finestret	Sep, 2010	13 (10+3)	8	0.62 (0.32–0.86)
5	France	Santa Maria Poggio	Jan, 2011	43 (25+18)	12	0.28 (0.15–0.44)
6	France	Mirabel	Aug, 2011	21 (15+6)	6	0.29 (0.11–0.52)
7	France	Bellegarde	Oct, 2012	17 (0+17)	0	0.00 (0.00–0.20)
8a	France	Mougins	Oct, 2012	13 (0+13)	6	0.46 (0.19–0.75)
8b	France	Mougins	Oct, 2013	28 (0+28)	16	0.57 (0.37–0.76)
9	France	Chaussan	Oct, 2013	10 (0+10)	3	0.30 (0.07–0.65)
10	France	Montpellier	Oct, 2013	19 (0+19)	11	0.58 (0.33–0.80)
11	France	Sauternes	Jan, 2014	30 (0+30)	21	0.70 (0.51–0.85)
12	France	Prigonrieux	Jan, 2014	44 (0+44)	16	0.36 (0.22–0.52)
13	France	Concourson sur Layon	Jun, 2014	8 (0+8)	4	0.50 (0.16–0.84)
14	France	Gotheron	Jul, 2014	17 (5+12)	6	0.35 (0.14–0.62)
15	France	Carrière sur Seine	Jan, 2014	35 (15+20)	8	0.23 (0.10–0.40)
16	France	Saint Germain d’Esteuil	Jan, 2014	22 (0+22)	8	0.36 (0.17–0.59)
17	Germany	Dossenheim	Oct, 2013	41 (0+41)	22	0.54 (0.37–0.59)
18	Italy	Vigolo Vattaro	Sep, 2013	16 (0+16)	16	1.00 (0.79–1.00)
19a	Italy	San Michele	Sep, 2013	9 (0+9)	2	0.22 (0.15–0.59)
19b	Italy	San Michele	Sep, 2014	20 (8+12)	13	0.65 (0.41–0.85)
20	Italy	Bari	Feb, 2014	11 (0+11)	8	0.73 (0.39–0.94)
21	Slovenia	Izola	Oct, 2013	35 (0+35)	16	0.46 (0.29–0.63)
22	Spain	Girona	Mar, 2014	37 (3+34)	29	0.85 (0.62–0.90)
23	Switzerland	Gottefrey	Oct, 2013	20 (0+20)	7	0.35 (0.15–0.59)

The sample number corresponds to the code of the sampling indicated in Fig 1. CI: 95% confidence interval.

### DNA extraction and *Wolbachia* detection by Polymerase Chain Reaction (PCR)

DNA from whole individuals was extracted using the Nucleo Spin Tissue kit (Macherey-Nagel). The quality of extraction was checked by PCR targeting the arthropod-specific locus Internal Transcribed Spacer *ITS2* gene (Its2U/Its2L) [32]. *Wolbachia* detection was performed using *Wolbachia* specific primers for two different genes: *ftsZ*, a bacterial cell division gene (F2/R2) [33], and *wsp* (*Wolbachia surface protein*) (81F/691R) [34]. PCR reactions were performed in 25 μL volumes containing 100 μM dNTP, 200 nM primers, 20mM Dream Taq Green Buffer, 0.5 IU *Taq* DNA polymerase (Eurobio) and 1μL of DNA template. Cycling conditions were 94°C (2 min), 94°C (30 sec), 55°C (*ITS2* and *ftsZ* primers) or 52°C (*wsp* primers) (30 sec), 72°C (45 sec), 72°C (10 min) for 34 cycles. All the primer sequences were indicated in S1 File. PCR products were visualized in 1% agarose gels. An individual was considered infected when the two *Wolbachia*-specific primers produced fragments of the appropriate size. To reduce the possibility of false negatives, all samples for which we failed to amplify *Wolbachia* signal with normal PCR were verified by a more sensitive real-time quantitative PCR method using the *wsp* primers 81F/691R. The 10μL reaction mixture contained 200 nM of each primer, 5 μL of Light Cycler^®^ 480 SYBR Green I Master (Roche) and 1 μL of DNA sample. The amplification consisted of 10 min at 95°C followed by 40 cycles of 10 sec at 95°C, 20 sec at 53°C and 30 sec at 72°C. Only for two individuals, *Wolbachia* infection was not detected by classic PCR but detected by real-time quantitative PCR.

### MLST approach

PCRs targeting six genes of *Wolbachia (wsp*, *gatB*, *coxA*, *hcpA*, *ftsZ*, *fbpA*) [34,35] were carried out on two randomly selected *w*Suz-infected samples per locality (Fig 1 and Table 1). All PCR reactions were performed as described before. Annealing temperature was 55°C except for *wsp* (52°C) and all the PCR products were sequenced. Sequences were aligned using MUSCLE [36] algorithm implemented in CLC DNA Workbench 6.9.1. (CLC Bio) and inspected by eye. On an average, we sequenced and compared approximately 3200 nucleotides per individual (between 470 and 610 nt per gene). All primers used in this study are presented in S1 File.

### Cytoplasmic Incompatibility (CI) assays

Two different populations of *D*. *suzukii* were used for CI experiments, one from France (Compiegne) and another from Italy (Valsugana). The French population was sampled in 2011 and reared in mass population in Lyon on a cornmeal diet (agar: 1%, dextrose: 8.75%, maize: 8.75%, yeast extract: 2%, nipagin: 3%) under constant lab conditions of 21±1°C temperature with a 12 hours light/dark cycle at 70% relative humidity. The Italian population was established from individuals collected in Valsugana region in 2011, and subsequently reared in laboratory at San Michele all’Adige on standard corn meal diet at a temperature of 23±1°C, 65% relative humidity with a 12:12 light/dark cycle. Maternal transmission of *Wolbachia* in the laboratory-established lines was perfect and thus the infection was stably maintained in the lab. Before starting the CI experiment, both lines were acclimatized under the same laboratory conditions for three generations in Cambridge Lab at a constant temperature of 22°C and 65% relative humidity with 12:12 light/dark cycle, thereby avoiding any bias due to lab environments.

To obtain *Wolbachia*-infected and uninfected fly lines with the same genetic background, antibiotic treatments were performed on infected lines using 0.25mg mL^-1^ tetracycline for three consecutive generations in mass populations. After treatments, flies were fed on normal standard diet for two further generations to recover them from any side effect caused by antibiotic treatment. Due to different lab practices, flies from Italian populations were further allowed to feed on natural fecal material from infected individuals to re-acquire their loss of gut-associated microbiota but flies from France were not gut-flora restored. Ten isofemale lines were established and the presence of *Wolbachia* was checked by PCR in mothers after laying eggs. The absence of *Wolbachia* was re-confirmed by real-time PCR as described above. This was repeated for three generations and then, for each population, one isofemale line was retained for crossing experiments. Their infection status was also checked and confirmed just before the CI assays (n = 20 for each line), which were performed on the 10^th^ generation after antibiotic treatments were stopped.

All types of crosses (infected female x infected male, infected female x uninfected male, uninfected female x infected male and uninfected female x uninfected male) were performed for each genetic background at Cambridge. Freshly hatched individuals were sexed and placed separately into cornmeal diet tubes to ensure the virginity of flies. A 3-days old virgin male and a 5-days old virgin female were allowed to mate in food vial for 24h. Females were then individually allowed to oviposit for 48h on grape-juice agar petri dish. Every 48h, females were transferred to a new petri dish, following 48h period for oviposition, and the total number of eggs along with the number of hatched larvae per female were recorded. Experiment was performed in replicates for each type of cross as mentioned in Table 2. The incompatibility relationship was determined by the rate of eggs that hatched. Females that laid less than 10 eggs were not included in the analysis (percentages are indicated in S2 File).

**Table 2:** Cytoplasmic incompatibility assays for *D*. *suzukii* from France and Italy.

Origin	Female	Male	N	Number of eggs laid	Mean hatch rate (±sd)
**France**	UN	UN	21	1113	0.691 (±0.041) b
	UN	IN	13	763	0.404 (±0.087) a
	IN	UN	15	588	0.573 (±0.075) ab
	IN	IN	15	795	0.718 (±0.069) b
**Italy**	UN	UN	28	1199	0.634 (±0.059) α
	UN	IN	38	2000	0.502 (±0.044) α
	IN	UN	25	1606	0.505 (±0.064) α
	IN	IN	27	1370	0.729 (±0.045) α

### Statistical analysis

We tested the difference in *Wolbachia* prevalence between American and European continents by fitting a generalized linear model with a quasi-binomial error (data for North America was taken from [19]). Samples originated from the same population but collected in several years were pooled. We tested for the existence of differences between samplings by using Fisher’s exact test. Estimates of the mean prevalence on each continent were transformed from logits into percentages. Statistical analysis of the CI data was done using a generalized linear model (quasi-binomial family). An ANOVA was done on this model and Tukey's (HSD) tests were used for two by two comparisons. R 3.2.2 version [37] was used to perform all the analysis.

### Ethics Statement

Sampling of *D*. *suzukii* was carried out on private lands with owners’ permission. All individuals were sampled in experimental stations and did not require specific permission. The field studies did not involve endangered or protected species.

## Results

### Variable *w*Suz infection frequencies in *D*. *suzukii* populations

We detected *Wolbachia* in 22 populations out of 23 surveyed from eight European countries (Fig 1). Out of the 561 *D*. *suzukii* flies tested, 258 were found infected with *Wolbachia* (Table 1). We found a highly variable infection prevalence (from 0 to 100%) between localities (χ^2^ = 192, d.f = 42, *P*<0.001). The two extreme values (0 and 100) have been observed only in two populations (Bellegarde in France and Vigolo Vattaro in Italy respectively); in all the other localities, prevalence ranged between 15% and 85% with a mean of 46%.

In two European localities, Mougins (in France) and San Michele (in Italy), we sampled for two consecutive years at the same period (October, 2012–13 and September, 2013–14 respectively) and found an increasing trend of the *Wolbachia* prevalence over time for both localities (Mougins: from 0.46 to 0.57; San Michele: from 0.22 to 0.65; Table 1) though the trend was not significant (Fisher’s exact test, *P* = 0.74 and *P* = 0.05 respectively).

Hamm [19] screened 929 individuals sampled from four American localities of *D*. *suzukii* over two years and found infection frequencies ranging from 7 to 58% with a mean of 17%. Therefore, in both continents, the prevalence of *w*Suz is highly variable. Overall, the European infection frequencies is found almost three times significantly higher than the ones in North America (GLM: *t* = 4.54, d.f = 26, *P*<0.001).

### European *D*. *suzukii* flies carry one single sequence type of *Wolbachia* “*w*Suz”

MLST analysis, assayed from two individuals per population (Fig 1 and Table 1), indicated that all European *D*. *suzukii* individuals carry the same *Wolbachia* sequence type (100% nucleotides identity for all genes). Sequences were identical to those of *w*Suz previously found and characterized in *D*. *suzukii* individuals originated from Italy [18] and North America [19]. The sequences obtained in the present study are recorded in Genbank as KS308222-7.

### Cytoplasmic incompatibility assays

To test whether *w*Suz is able to induce CI, which could facilitate its spread throughout European *D*. *suzukii* populations, we performed mating experiments on flies originating from France and Italy (Table 2). In diploid species, CI leads to the death of embryos when infected males mate with uninfected females; therefore in case of *w*Suz induced CI expression, we expected a lower egg hatch rate in these crosses. We found a significant effect of the type of cross on the hatching rates only for the French population (F = 3.86, d.f = 3, *P* = 0.01; for Italian populations: F = 2.14, d.f = 3, *P* = 0.10). Globally, the hatching rate was higher in crosses that involved individuals of the same infection status (control crosses) than that of different infection status (infected and uninfected). The “incompatible” crosses (infected male x uninfected female) tended to have the lowest hatching rate. For the French population, the difference was significant only when the incompatible cross was compared to the two control crosses: infected male and female (Tukey HSD test, *P* = 0.02) and uninfected male and uninfected female (Tukey HSD test, *P* = 0.01). For the Italian population there is no significant difference between the incompatible cross and the three others. Therefore, like in the American populations, these results do not allow us to conclude that *w*Suz can induce CI in European *D*. *suzukii* populations.

Mean eggs hatch rate (only females that lay at least 10 eggs) ± standard deviation (sd). N: number of crosses. IN: infected with *Wolbachia*, UN: uninfected. Statistical analysis was performed on lines from the two origins separately. One-way ANOVA indicated that the hatching rate differed significantly between the mating types. One-way ANOVA was followed by Tukey’s test to compare between crossing types (means marked with the same letter are not significantly different; *P* = 0.05).

## Discussion

We found that the *Wolbachia* infection in *D*. *suzukii* is highly variable in European populations with a mean prevalence of 46%. This infection rate differs significantly from the one found in the North America (17% in average) [19]. Several parameters could explain this variability such as the efficiency of vertical transmission or the costs/benefits of infection, which might differ between continents. Moreover, variability can also be due to differences in the host and/or *Wolbachia* genetic backgrounds as indicated in other insect species by trans-infection experiments using a given *Wolbachia* strain injected into different hosts (for example, see [38]). Apart from different host-*Wolbachia* association dynamics, the variability can also be dependent on environmental factors (*i*.*e*. temperature, diet, larvae density…) [39,40,28,41,42] that might vary locally and across time with their colonization dynamics, hence providing higher fitness advantage for *Wolbachia* persistence in European *D*. *suzukii* populations than in the American ones. If this is true, we should expect persistence at most/all localities to a certain infection rate representing the fitness advantage based on that particular locality’s environmental and habitat specificity. Although we found a (non-significant) increasing trend for the *Wolbachia* prevalence in two localities, more sampling time points over longer durations are needed to draw a clearer picture.

Infection dynamics of vertically transmitted symbionts depend on the phenotypic effect induced on the host, the fitness cost and/or benefits of the infection, and how reliably symbionts are inherited. Our results clearly showed that *w*Suz is not fixed in *D*. *suzukii* populations as otherwise expected in cases of obligatory mutualism. We did not detect any significant evidence for CI induction by *w*Suz in Italian or French *D*. *suzukii* population. An imperfect vertical transmission of endosymbiotic bacteria (20 to 95% in American populations) [19] associated with an absence of strong reproductive manipulation would theoretically lead to elimination of the symbiont from host populations unless it provides the other host fitness advantages [29]. The stable presence of *w*Suz in all but one of the populations studied in America and Europe would thus suggest that *w*Suz may provide direct benefits to its host allowing its maintenance in natural populations of *D*. *suzukii*. Another possibility would be frequent intraspecific horizontal transfers of *Wolbachia* [43]. However, we favored the first hypothesis because the second one would imply that horizontal transfers occur in all populations from two continents, which seems unlikely. In addition, *Wolbachia* are also known as a mutualist in insects [44] providing beneficial effects to their native hosts, such as an increase of host survival [45] or fecundity under nutritional stress [46], or protection against viral, microbial, and fungal pathogens [47,48,49,50,51,52]. While no fitness benefits have been detected in American populations [19], a recent study suggested a beneficial effect of *Wolbachia* infection on female fecundity in an Italian population of *D*. *suzukii* [53]. Since both of the studies used different protocols to perform the assays, different lab practices might impair the significance of this difference. For example, Hamm [19] crossed native *Wolbachia* infected and uninfected flies from the wild: thereby we cannot exclude the confounding effect of different host genetic backgrounds on the fly fecundity. On the other hand, Mazzetto [53] compared infected individuals to uninfected ones that were immediately obtained after antibiotic treatments. Therefore the reduced fecundity of uninfected females can also be explained by side effects of the antibiotics on mitochondria and/or host metabolism [54]. Although with caution, excluding any of these confounding factors suggest that the beneficial effect induced by *w*Suz may depend on the fly genetic background and/or that *D*. *suzukii* from Europe and North America are infected by different *Wolbachia* strains. Highly sophisticated approaches, for example, Next Generation Sequencing (NGS) based tools are needed to have more insights into the *Wolbachia* strain diversity in *D*. *suzukii* populations. Moreover, future experiments involving exchange or introgression of European and American *Wolbachia* strains to study the life history traits of host organisms in the presence and absence of *Wolbachia* would help to unveil whether differences in *w*Suz-mediated fitness benefits in different continental *D*. *suzukii* populations are *Wolbachia* strain-dependent or host dependent.

There is a growing interest in exploring symbiotic microorganisms for biocontrol management programs [55,56,57]; in the case of *Wolbachia*, the idea is to exploit its capability to induce CI to control natural populations of arthropod pests in an Incompatible Insect Technique (IIT) fashion [58,59], where males infected with a CI-inducing strain of *Wolbachia* are released in the field to mate with incompatible females leading to embryo mortality and ultimate population suppression. This procedure is somewhat analogous to the Sterile Insect Technique (SIT), a species-specific method of insect control that relies on the release of large numbers of sterile males instead of incompatible males [60], but the advantage is that insects do not have to be irradiated or genetically modified before release. The inconvenience is, however, that IIT and SIT require solid methods allowing efficient males and females separation, but *D*. *suzukii* males are easily recognizable because of their spotted wings [1]. Considerable research efforts have already demonstrated that *Wolbachia*-inducing CI could be used as a tool for population control [61,62,63]. However, it might be more complicated if *Wolbachia* naturally infects the insect pest like in the case of *D*. *suzukii* since it would require finding an incompatible CI-inducing *Wolbachia* strain, which is not rescued by *w*Suz in its original host. Hamm [19] and our results are concordant in showing that *D*. *suzukii* is infected by only one strain of *Wolbachia*, which is present in almost all populations screened and does not induce CI. Trans-infection experiments using closely related *Wolbachia* strains such as the highly CI-inducing *w*Ri strain from *D*. *simulans* will thus be needed to assess modification and rescue capabilities of different strain combinations towards a bidirectional CI-based control strategy.

## Supporting Information

S1 File Primers used in this study. (PDF)

S2 File Percentage of excluded females that laid less than 10 eggs in crosses experiment. (PDF)
