# Correction: *Wolbachia* in European Populations of the Invasive Pest *Drosophila suzukii*: Regional Variation in Infection Frequencies

**DOI:** 10.1371/journal.pone.0150050

**Published:** 2016-02-19

**Authors:** Julien Cattel, Rupinder Kaur, Patricia Gibert, Julien Martinez, Antoine Fraimout, Francis Jiggins, Thibault Andrieux, Stefanos Siozios, Gianfranco Anfora, Wolfgang Miller, Omar Rota-Stabelli, Laurence Mouton

The image for Fig 1 is incorrect. Please see the corrected Fig 1 here.

**Fig 1:**
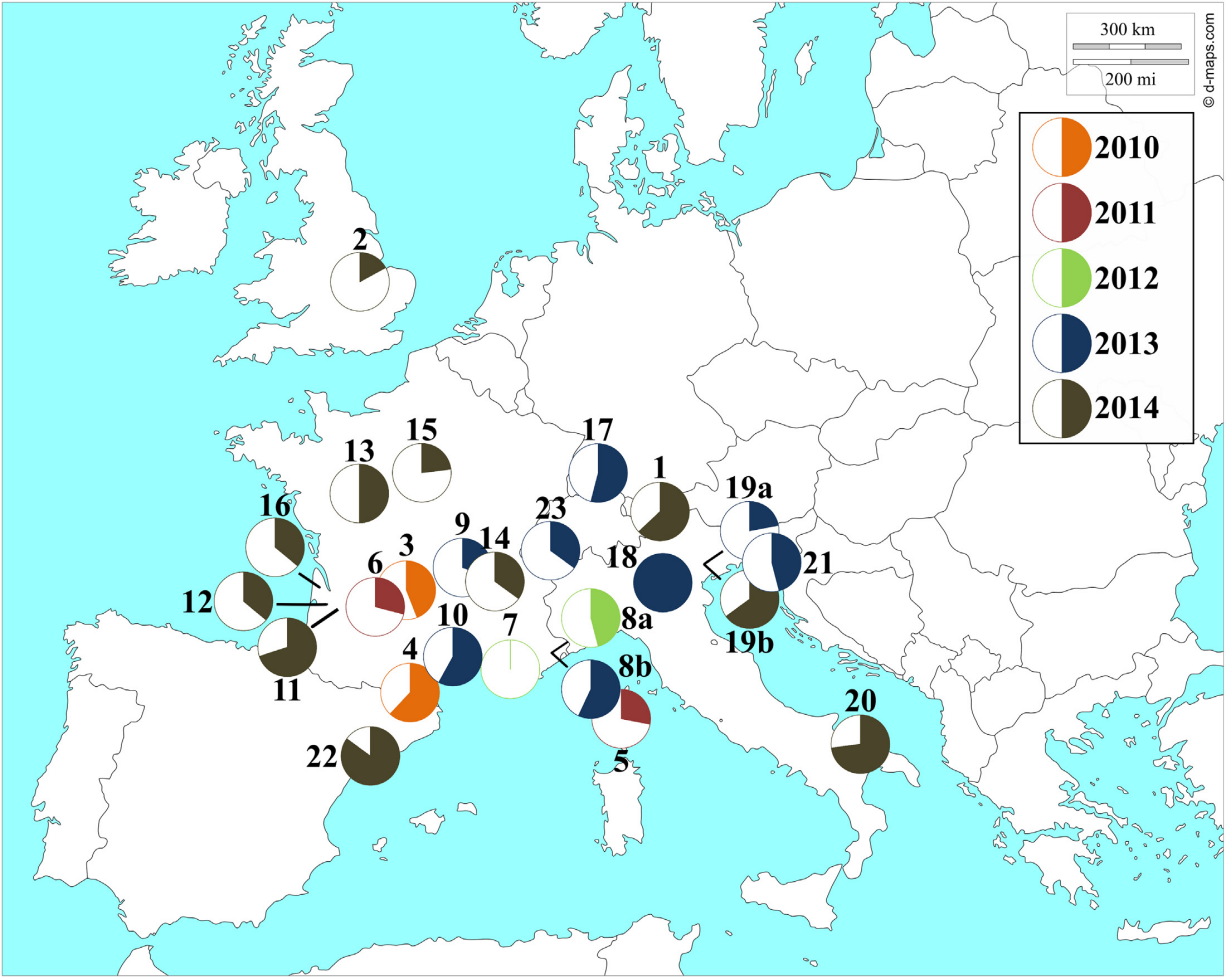
*Wolbachia* prevalence and collection sites for *D*. *suzukii* individuals. A number was assigned for each sampling site and details for each locality are given in the Table 1. Reprinted from http://d-maps.com under a CC BY license, with permission from Daniel Dalet, original copyright 2007–2016.
