# Supplementary material for: Wolbachia in European Populations of the Invasive Pest Drosophila suzukii: Regional Variation in Infection Frequencies
Source: PLoS One. 2016 Jan 25;11(1):e0147766. doi: 10.1371/journal.pone.0147766 (PMC4725738; doi:10.1371/journal.pone.0147766)
Supplement: S1 File — (PDF) [file pone.0147766.s001.pdf]

Gene target	Primer name	Sequence (5'-3')	fragment size (bp)	Annealing temperature (°C)
wsp	81F	TGGTCCAATAAGTGATGAAGAAAC	610	52
	691R	AAAAATTAAACGCTACTCCA		
ftsZ	ftsZ-F2	TTGCAGAGCTTGGACTTGAA	480	55
	ftsZ-R2	CATATCTCCGCCACCAGTAA		
Its2	Its2U	TGTGAACTGCAGGACACATG	500	55
	Its2L	AATGCTTAAATTTAGGGGGTA		
coxA	coxA-F1	TTGGRGCRATYAACTTTATAG	486	55
	coxA-R1	CTAAAGACTTTKACRCCAGT		
gatB	gatB-F1	GAKTTAAAYCGYGCAGGBGTT	470	55
	gatB-R1	TGGYAAYTCRGGYAAAGATGA		
hcpA	hcpA-F1	GAAATARCAGTTGCTGCAAA	514	55
	hcpA-R1	GAAAGTYRAGCAAGYTCTG		
ftsZ	ftsZ-F1	ATYATGGARCATATAAARGATAG	629	55
	ftsZ-R1	TCRAGYAATGGATTRGATAT		
fbpA	fbpA-F1	GCTGCTCCRCRRGGYWTGAT	508	55
	fbpA-R1	CCRCCAGARAAAAYYACTATTC		
