# Supplementary material for: Wolbachia in European Populations of the Invasive Pest Drosophila suzukii: Regional Variation in Infection Frequencies
Source: PLoS One. 2016 Jan 25;11(1):e0147766. doi: 10.1371/journal.pone.0147766 (PMC4725738; doi:10.1371/journal.pone.0147766)
Supplement: S2 File — (PDF) [file pone.0147766.s002.pdf]

Origin	Female	Male	Total no. of crosses setup<br><br>N	Number of females laid <10 eggs	Percentage of females excluded from crosses<br><br>%
**France**	UN	UN	31	10	32.25
	UN	IN	17	4	23.52
	IN	UN	23	8	34.78
	IN	IN	23	8	34.78
**Italy**	UN	UN	28	0	0
	UN	IN	41	3	7.31
	IN	UN	33	8	24.24
	IN	IN	33	6	18.18
